# The Effect of Physical Training on Peripheral Blood Mononuclear Cell Ex Vivo Proliferation, Differentiation, Activity, and Reactive Oxygen Species Production in Racehorses

**DOI:** 10.3390/antiox9111155

**Published:** 2020-11-20

**Authors:** Olga Witkowska-Piłaszewicz, Rafał Pingwara, Anna Winnicka

**Affiliations:** 1 Department of Pathology and Veterinary Diagnostics, Institute of Veterinary Medicine, Warsaw University of Life Sciences—SGGW, 02-776 Warsaw, Poland; anna_winnicka@sggw.edu.pl; 2 Department of Physiological Sciences, Institute of Veterinary Medicine, Warsaw University of Life Sciences—SGGW, 02-776 Warsaw, Poland; rafal_pingwara@sggw.edu.pl

**Keywords:** lymphocytes, monocytes, reactive oxygen species (ROS), exercise, anti-inflammatory state, pro-inflammatory state, Tregs, cytokines

## Abstract

Physical activity has an influence on a variety of processes in an athlete’s organism including the immune system. Unfortunately, there is a lack of studies regarding racehorse immune cells, especially when the horse model is compared to human exercise physiology. The aim of the study was to determine changes in immune cell proliferation, lymphocyte populations, and monocyte functionality in trained and untrained racehorses after exercise. In this study, field data were collected. The cells from 28 racehorses (14 untrained and 14 well-trained) were collected before and after exercise (800 m at a speed of about 800 m/min) and cultured for 4 days. The expression of CD4, CD8, FoxP_3_, CD14, MHCII, and CD5 in PBMC, and reactive oxygen species (ROS) production, as well as cell proliferation, were evaluated by flow cytometry. In addition, IL-1β, IL-4, IL-6, IL-10, IL-17, INF-γ, and TNF-α concentrations were evaluated by ELISA. The creation of an anti-inflammatory environment in well-trained horses was confirmed. In contrast, a pro-inflammatory reaction occurred in untrained horses after training. In conclusion, an anti-inflammatory state occurs in well-trained racehorses, which is an adaptational reaction to an increased workload during training.

## 1. Introduction

In humans, there are studies that include both the innate and adaptive immune response after exercise, but the detailed influence on the whole body is still unknown [1,2,3]. Exhaustive and prolonged exercise can result in a transient decrease of leukocyte functions which is linked with reduced T, B, and natural killer (NK) cell activity in humans [3]. This short-term window of immune suppression is termed the “open-window” hypothesis. It results in increased viral infections of the upper respiratory tract (URTI) during recovery time which was documented both in humans [4,5] and horses [6,7]. Conversely, long term physical activity reduces the risk of chronic inflammatory disorders, viral and bacterial infections [8,9]. Exercise immune-enhancement during training is obtained by redeploying immune cells to peripheral tissues, where leucocytes can recognize and destroy damaged/infected cells [10,11]. Thus, it is not clear if the occurrence of immune system reactions after exercise is a positive adaptation or is harmful. 

Unfortunately, there are a limited number of studies concerning the effect of exercise on racehorse immune cells. Racing is an equestrian sport of great popularity, in which horses are introduced to intensive training at a very young age (1–2 years). This makes these animals susceptible to infections due to overtraining. In horses, most studies have focused on the redistribution of white blood cells such as monocytes, neutrophils, and lymphocytes after low-intensity long-duration exercise [7,12]. Physical activity induces leukocytosis and cells experience transient changes which depend on exercise intensity and duration [3,13]. It is mainly caused by hemoconcentration resulting from fluid shifts and changes in catecholamine and cortisol levels. However, white blood cell numbers return to a baseline level in approximatively 1 h after the end of exercise [14].

There are also studies measuring the response of the innate immune system by determining phagocytic and/or oxidative burst activity of neutrophils and monocytes [13,15]. However, there is a lack of studies regarding the monocyte phenotype and functions. In addition, the effect of exercise on the adaptive immune response was not evaluated. Only lymphocyte proliferation and cytokine production were measured [15,16]. There is only one study with a limited number of horses where the number of CD4+ T regulatory cells (Treg) was shown to decrease after the completion of a 50-mile endurance ride, with no change in cytotoxic lymphocyte T CD8+ (Tc) numbers [15]. Moreover, there are no studies regarding the forkhead box P_3_ (FoxP_3_) expression, which is a key transcription factor that governs the maturation of Treg cells [17]. Thus, more knowledge of changes in the immune system due to training is crucial to prevent illnesses and to maintain horse welfare.

The aim of the study was to determine changes in immune cell proliferation, lymphocyte populations, and monocyte functionality in trained and untrained racehorses after exercise.

## 2. Materials and Methods

### 2.1. Animals and Blood Sampling

In this study, field data was collected. The study involved 28 healthy 2–4 year-old racehorses of both genders (50% male, 50% female). Horses were divided into two groups: 14 well-trained (WT) after 1–2 training seasons with good performance (3–4 years old) and 14 untrained (UT) thoroughbreds (2–3 years old) at the beginning of their race training. They were fed with the standard diet designed for racehorses (oats 5.5 kg/horse, meadow hay 7.5 kg/horse and special concentrate for performance horses). The concentrated feed and roughage were given three times per day. All horses were trained by one trainer and housed in the same stable on straw under the same environmental conditions. The heart rate, mucous membranes (color and moisture), capillary refill time and dehydration (measured as the time it takes for a pinched skin fold over the point of the shoulder to flatten) were measured by the veterinary practitioner before and after exercise. Thus, basic clinical examinations before and after training revealed no clinical symptoms of any disease. The training sessions were performed on days of similar temperature (18 °C ± 1.0) and humidity (45% ± 0.8) to avoid weather influence and changes in the racetrack. The horses exercised on sand for 800 m at a speed of about 800 m/min. For untrained horses, it was the first training session with a gallop. 

Blood samples were acquired by jugular venipuncture at rest, approximately 1 h before feeding and 30 min after the training session using the BD Vaccutainer system (BD, Franklin Lakes, NJ, USA) with heparin tubes for peripheral blood mononuclear cell (PBMC) isolation. All samplings were part of standard veterinary diagnostic procedures, and according to the Polish legal regulations; approval of the Local Commission for Ethics in Animal Experiments was not required. [18]

### 2.2. Cell Isolation and Culture

PBMCs were isolated from heparinized blood by density gradient centrifugation (SepMate™—Lymphoprep™ System, Cologne, Germany). Following density gradient isolation, PBMCs were washed twice in 2% BSA in PBS. All cell cultures were performed in RPMI 1640 medium with GlutaMAX™ containing 10% heat-inactivated horse serum, penicillin (100 IU/mL), streptomycin (100 μg/mL), nonessential amino acids (1%), MEM vitamins (100 μM), sodium pyruvate (1 mM), and amphotericin B (1 μg/mL) (all reagents were purchased from Gibco™, Life Technologies, Bleiswijk, Netherlands). 4 × 10^6^ freshly isolated PBMCs were cultured in the absence or presence of a phytohemagglutinin (PHA) (Sigma-Aldrich, St. Louis, MO, USA; 5 μg/mL). Samples designated for the determination of the cell proliferation were supravitally stained with CellTrace™ Violet Cell Proliferation Kit (Life Technologies, Bleiswijk, The Netherlands) before culture according to the manufacturer’s instructions. After 24 h cells were washed and recombinant equine IL-2 (R and D Systems, Abingdon, UK; 100 U/mL) was added and the cells were incubated for another 3 days. All cells were incubated at 37 °C with 5% CO_2_.

### 2.3. Cell Staining

After 4 days the production of reactive oxygen species by isolated PBMCs was measured using CellRox (CR) Deep Red Assay Kit (Life Technologies, Paisley, Scotland) according to the manufacturer’s protocol. Tert-butyl hydroperoxide solution (TBHP) was used as an inducer of reactive oxygen species (ROS) production. To minimalize the risk of cell damage and to obtain adhesive cells, Corning^®^ Cellstripper^®^ Solution (Mediatech, Inc., Manassas, VA, USA) was used according to manufacturer’s instructions. For the analysis of lymphocytes, only non-adherent cells were collected, as well as for the determination of the cell proliferation. PBMCs were characterized by checking the expression of the surface markers using equine specific antibodies or with documented cross-reactivity included in Table 1. The appropriate amount and concentration of each antibody have been determined empirically to obtain optimal labelling results. The controls included unlabeled cells, and when necessary, the FMO (fluorescence minus one) and “switch-off” approach (SWOFF) controls were performed. The cells were incubated with antibodies for 20 min at 4 °C in eBioscience™ Flow Cytometry Staining Buffer (Life Technologies, Bleiswijk, The Netherlands) in the dark. The cells were then washed twice with 2% BSA and resuspended in 200 μL flow cytometry staining buffer and immediately introduced into the cytometer. For FoxP_3_ staining eBioscience™ FoxP_3_/Transcription Factor Staining Buffer Set (Life Technologies, Bleiswijk, The Netherlands) was used according to the manufacturer’s protocol.

### 2.4. Flow Cytometry Analysis

**Figure 1:**
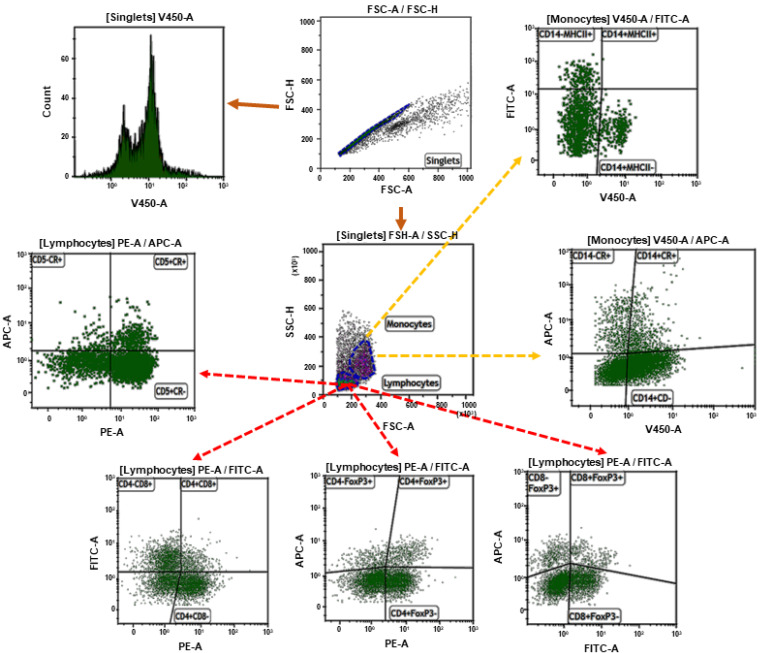
The gating strategy. Doublets were removed from the analysis by setting the gate on single cells on the FSC-area (FSC-A) vs. FSC-high (FSC-H) dot plot. Cell proliferation was calculated from singlets. Next, the lymphocytes or monocytes were gated based on FSC and SSC dot plots. Then the gate included lymphocytes and analysis of CD4+, CD8+, and FoxP_3_ cells were made. The second sample included CD5+, CD14+, MHCII+ cells, and the median fluorescence intensity (MFI) of ROS was calculated on that cell population.

The flow cytometric analysis was performed using a FACSCanto II flow cytometer and Kaluza 1.5. software (Beckman Coulter, Brea, CA, USA); 10,000 cells of each sample were acquired. Prior to multicolor staining, the compensation was set using single-positive cells for each color. The gating strategy has been shown in Figure 1.

### 2.5. ELISA

The PBMCs’ cytokines production was measured by obtaining their concentration in the supernatant after 4 days of cell culturing. The supernatant was obtained after 300× *g* 10 min centrifugation and was stored at −80 °C. The levels of IL-1β, IL-4, IL-6, IL-10, IL-17, INF-γ, and TNF-α were determined by commercially available immunoenzymatic commercial assays dedicated for equine species, according to the manufacturer’s protocols (ELISA kit, BT Lab, Shanghai, China). The absorbance was measured by Multiscan Reader (Labsystem, Helsinki, Finland) using a Genesis V 3.00 software program. All samples were tested in duplicate.

### 2.6. Data Enumeration and Statistical Analysis

The statistical analysis was performed in Prism software, version 5.0 (GraphPad Software, San Diego, CA, USA). To determine the statistical significance of the differences in the examined parameters in each UT and WT horse before vs. after training, the Wilcoxon signed rank test was used. The comparison between the differences of the examined parameters in untrained and trained horses was calculated according to the formula:

difference = % or MFI of cells after training − % or MFI of cells before training,(1)

The comparisons were done with the use of the Student’s *t*-test for unpaired groups. Numerical variables were presented as the arithmetic mean ± standard error of the mean (SEM). The significance level was indicated as follows: * *p* < 0.05; ** *p* < 0.01; *** *p* < 0.001.

## 3. Results 

### 3.1. Lymphocyte Proliferation

To determine the lymphocyte proliferation intensity, Cell Trace Violet staining was used. The data obtained are inversely proportional to the proliferative activity because with each cell division the fluorescence intensity of dye is lower. The data were shown as the reciprocal of the MFI value (1/MFI). 

**Figure 2:**
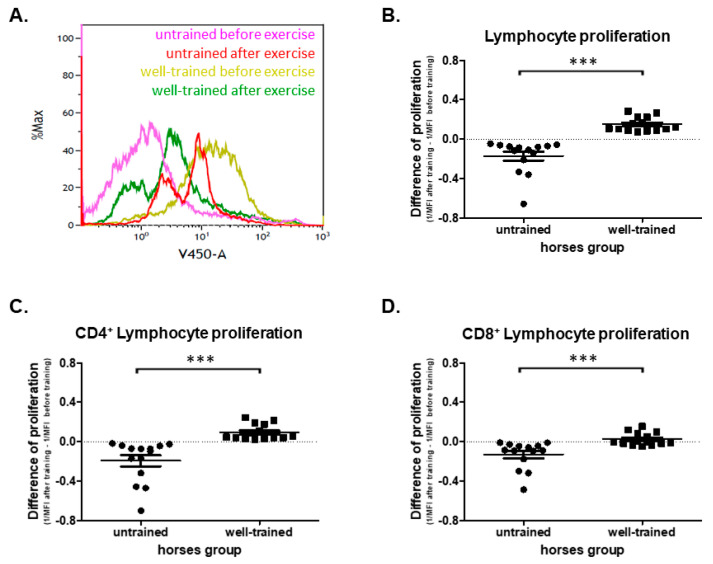
(**A**) Representative histogram showing T lymphocyte proliferation obtained before and after exercise from untrained and well-trained horses. The graph shows total (**B**), CD4+ (**C**), and CD8+ (**D**) lymphocyte proliferation. The results are presented as the mean ± SEM. Significance levels are: *** *p* < 0.001.

The study showed a statistically significant decrease (*p* = 0.0001) in the harvested cells’ proliferation activity after training (0.203 ± 0.0629) in comparison to cells harvested before training (0.375 ± 0.0260) from UT horses (Supplementary Figure S1A). The opposite impact was observed in WT horses in which a highly significant increase (*p* = 0.0001) in the cell proliferation before training (0.0811 ± 0.0045) compared to those after training (0.1500 ± 0.0193) was noted (Supplementary Figure S1A). Moreover, a difference between lymphocyte proliferation between UT (−0.171 ± 0.0459) and WT horses after exercise (*p* < 0.0001) was found (−0.150 ± 0.0193) (Figure 2B). Detailed analysis confirmed that the total lymphocyte proliferation activity directly resulted in both CD4+ and CD8+ lymphocyte proliferation. The effect of exercise on the change in proliferation rate between UT vs. WT was observed in both CD4+ (*p* < 0.0001; −0.194 ± 0.0565 vs. 0.0945 ± 0.0210) and CD8+ (*p* = 0.0009; −0.131 ± 0.0377 vs. 0.0244 ± 0.0172) (Figure 2C,D). In UT horses, the inhibitory effect of exercise (before vs. after) on CD4+ (*p* = 0.0011; 0.421 ± 0.0810 vs. 0.227 ± 0.0326) and CD8+ (*p* = 0.0001; 0.292 ± 0.0505 vs. 0.162 ± 0.0192) cell proliferation was demonstrated (Supplementary Figure S1B,C). The opposite effect was observed in WT horses on both CD4+ (*p* = 0.0001; 0.0988 ± 0.00719 vs. 0.1760 ± 0.02080) and CD8+ (*p* = 0.0006; 0.0729 ± 0.00363 vs. 0.1230 ± 0.01600) cell proliferation (Supplementary Figure S1B,C).

### 3.2. T Lymphocyte Phenotype

**Figure 3:**
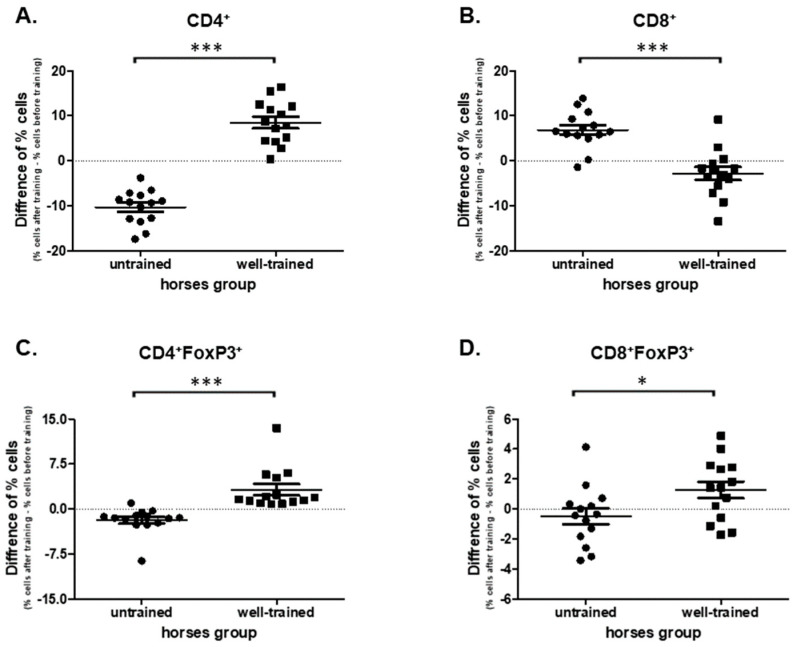
Graphic representation showing percentages of positive cells: CD4+ (**A**), CD8+ (**B**), CD4+FoxP_3_+ (**C**), and CD8+FoxP_3_+ (**D**) gated from total lymphocytes. The results are presented as the mean ± SEM. Significance levels are: * *p* < 0.05; *** *p* < 0.001.

Comparing the cells harvested from the UT horses before and after exercise a significant decrease in CD4+ (*p* = 0.0001; 36.60 ± 1.040 vs. 26.63 ± 0.884) and an increase in CD8+ (*p* = 0.0004; 31.70 ± 0.650 vs. 38.50 ± 1.130) percentages were observed (Supplementary Figure S2A,B). The opposite effect was found in the WT horses. An increase of CD4+ (*p* = 0.0001; 23.70 ± 1.050 vs. 32.10 ± 1.310) and a decrease of CD8+ (*p* = 0.0353; 35.1 ± 1.490 vs. 32.3 ± 0.366) percentages of cells were noted (Supplementary Figure S2A,B). In addition, statistically significant differences (*p* < 0.0001) in the change of CD4+ and CD8+ percentages under exercise were observed in cells obtained from UT (−10.3 ± 1.020; 6.86 ± 1.110; CD4+, and CD8+, respectively) and WT (8.49 ± 1.280; −2.81 ± 1.440; CD4+, and CD8+, respectively) horses (Figure 3A,B).

The statistically significant impact of the exercise (before vs. after) on the percentages of CD4+FoxP_3_ and CD8+FoxP_3_ regulatory cells was observed in both groups. In UT horses there was a decrease (*p* = 0.0006; 5.39 ± 0.527 vs. 3.55 ± 0.324), whereas in WT horses an increase (*p* < 0.0001; 4.68 ± 0.299 vs. 7.90 ± 0.860) in the percentages of CD4+FoxP_3_ cells (Supplementary Figure S2C,D). No effect of exercise was observed in the percentages of CD8+FoxP_3_ cells in the UT group (*p* = 0.3258; 3.85 ± 1.43 vs. 3.36 ± 1.84). However, an increase in CD8+FoxP_3_ cells in WT horses was noted (*p* = 0.0494; 3.85 ± 0.832 vs. 5.13 ± 2.230) (Supplementary Figure S2C,D). The analysis of the exercise impact on the T regulatory cells subpopulations showed differences between UT and WT horses in CD4+FoxP_3_ (*p* < 0.0001; −1.84 ± 0.582 vs. 3.22 ± 0.930), and CD8+FoxP_3_ (*p* = 0.0296; −0.486 ± 0.531 vs. 1.28 ± 0.552) cell subpopulations (Figure 3C,D).

### 3.3. Monocyte Phenotypes

**Figure 4:**
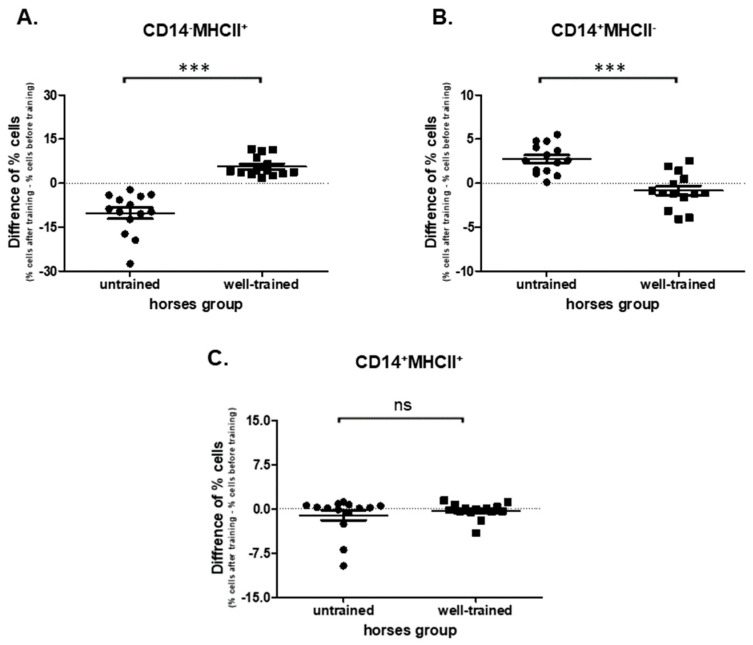
Graphic representation showing percentages of positive cells: CD14-MHCII+ (**A**), CD14+MHCII− (**B**), and CD14+MHCII+ (**C**) gated from total monocytes. The results are presented as the mean ± SEM. Significance levels are: *** *p* < 0.001.

The significant impact of the exercise (before vs. after) on CD14-MHCII+ cell populations in both UT (*p* = 0.0001; 21.8 ± 1.50 vs. 11.6 ± 1.030) and WT (*p* = 0.0001; 12.3 ± 2.040 vs. 18.0 ± 2.000) horses was confirmed (Supplementary Figure S3A). Significant (*p* < 0.0001) changes of percentages of CD14-MHCII+ cells between UT (−10.2 ± 1.880) and WT (5.68 ± 0.931) horses were observed (Figure 4A). Increase of CD14+MHC-cells in UT (*p* = 0.0001; 5.39 ± 0.619 vs. 8.13 ± 0.784) and no change in WT horses (*p* = 0.1937; 7.19 ± 0.778 vs. 6.34 ± 0.810) were detected (Supplementary Figure S3B). Analysis of exercise impact (before vs. after) on the CD14+MHCII+ cell subpopulations showed no difference in both UT (*p* = 0.8753; 1.74 ± 0.808 vs. 0.638 ± 0.1230) and WT (*p* = 0.4973; 1.840 ± 0.749 vs. 1.510 ± 0.492) horses (Supplementary Figure S3C). In addition, there was no change (*p* = 0.4139) of percentages of CD14+MHCII+ cells in UT (−1.11 ± 0.861) in comparison to WT (−0.330 ± 0.362) horses (Figure 4C).

### 3.4. ROS Production

**Figure 5:**
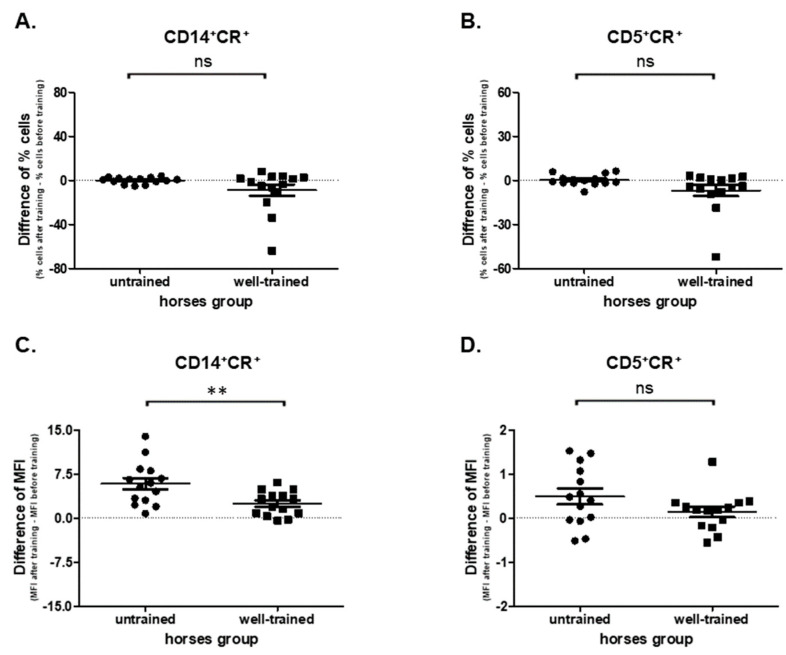
Graphic representation showing percentages of positive cells: CD14+CR+ (**A**), CD5+CR+ (**B**), and CD14+MHCII+ gated from total lymphocytes/monocytes and median fluorescent intensity of CR (CellRox) in CD14+ (**C**) and CD5+ (**D**) cells. The results are presented as the mean ± SEM. Significance levels are: ** *p* < 0.01.

In the present study no effect of exercise (before vs. after) on the CD14+CR+ cells’ percentages was observed in UT (*p* = 0.7148, 3.77 ± 0.586 vs. 3.88 ± 0.507) and WT (*p* = 0.1726, 18.60 ± 5.000 vs. 9.84 ± 1.110) horses (Supplementary Figure S4A). In addition, no statistically significant differences (*p* = 0.1005) in CD14+CR+ cells’ percentages between UT (0.102 ± 0.755) and WT (−8.760 ± 5.150) horses were observed (Figure 5A). However, statistically significant differences were found (*p* = 0.0062) in ROS (CR MFI) production between UT (5.86 ± 0.986) and WT (2.49 ± 0.559) horses (Figure 5C). The increase in CR MFI was observed in both UT (*p* = 0.0001; 9.61 ± 1.040 vs. 15.5 ± 1.730) and WT (*p* = 0.0009; 5.55 ± 0.596 vs. 8.04 ± 0.874) horses upon exercise (before vs. after) (Supplementary Figure S4C).

A reduced influence of exercise (before vs. after) on the CD5+CR+ cells’ percentages in WT (*p* = 0.0494; 10.9 ± 3.560 vs. 4.14 ± 0.586) and UT (*p* = 0.9032; 6.71 ± 0.875 vs. 6.98 ± 1.100) horses was noted (Supplementary Figure S4B). No statistically significant change was found (*p* = 0.0863) in CD5+CR+ cells between UT (0.0269 ± 0,995) and WT (−6.79 ± 3.83) horses (Figure 5B). The intensity of ROS production by CD5+ cells was increased after exercise in UT (*p* = 0.0353; 2.67 ± 0.194 vs. 3.16 ± 0.224), but not in WT (*p* = 0.2718; 3.39 ± 0.263 vs. 3.53 ± 0.298) horses (Supplementary Figure S5D). Nevertheless, no differences (*p* = 0.1197) in this examined parameter were observed between UT (0.489 ± 0.182) and WT (0.140 ± 0.118) horses (Figure 5D).

### 3.5. Cytokine Production

This study evaluated the main pro- and anti-inflammatory cytokine secretion ex vivo by PBMCs obtained from UT and WT horses before and after exercise. The obtained data confirmed the creation of an anti-inflammatory state and pro-inflammatory activity in WT and UT horses, respectively, under the influence of exercise.

A statistically significant increase (before vs. after) in pro-inflammatory cytokines’ concentration such as IL-1β (*p* = 0.0195; 20.6 ± 3.230 vs. 29.1 ± 3.280), IL-17 (*p* = 0.0039; 88.7 ± 40.400 vs. 122 ± 52.600), TNF-α (*p* = 0.0078; 76.1 ± 6.270 vs. 102 ± 7.480) and no statistically significant differences in the IFN-γ (*p* = 0.3008; 97.5 ± 1.730 vs. 108 ± 4.450) were noted in UT horses (Supplementary Figure S5A,E,F,G). In WT horses a significant decrease in pro-inflammatory cytokines’ concentrations such as IL-17 (*p* = 0.0039; 220.0 ± 11.800 vs. 36.7 ± 2.2250), and IFN-γ (0.0039; 127.0 ± 3.280 vs. 116.0 ± 1.800) and no change in IL-1β (*p* = 0.9102; 34.0 ± 3.120 vs. 34.0 ± 2.080) and TNF-α (*p* = 0.4961; 89.1 ± 4.800 vs. 92.2 ± 6.450) were observed (Supplementary Figure S5A,E,F,G).

**Figure 6:**
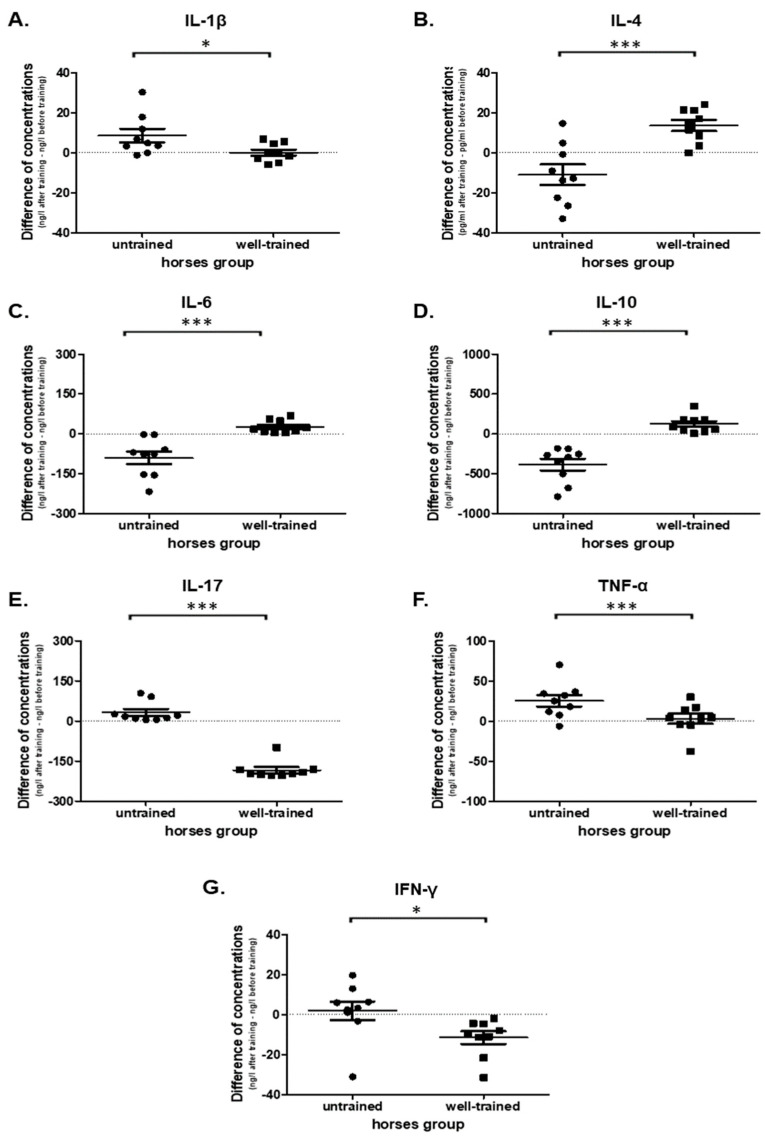
Graphic representation showing the cytokine concentrations: IL-1β (**A**), IL-4 (**B**), IL-6 (**C**), IL-10 (**D**), IL-17 (**E**), TNF-α (**F**), and IFN-γ (**G**) presented in culture medium of PBMCs obtained from untrained and well-trained horses. The results are presented as the mean ± SEM. Significance levels are: * *p* < 0.05; *** *p* < 0.001.

However, cytokine production showed exercise-related changes in each examined proinflammatory factor and differences between UT and WT horses: IL-1β (*p* = 0.0345; 8.55 ± 3.360 vs. 0.0178 ± 1.520), IL-17 (*p* < 0.0001; 33.0 ± 12.700 vs. −184 ± 11.100), TNF-α (*p* = 0.0319; 25.6 ± 7.290 vs. 3.03 ± 6.280), and IFN-γ (*p* = 0.0305; 1.87 ± 4.710 vs. −11.6 ± 3.150) (Figure 6A,E,F,G,). The IL-6 concentration under exercise (before vs. after) decreased in UT (*p* = 0.0039; 128 ± 22.700 vs. 37.6 ± 3.740) and increased in WT (*p* = 0.0039; 37.1 ± 64.3 ± 4.270) horses (Supplementary Figure S5C). Moreover, a significant difference (*p* = 0.0003) in IL-6 secretion was observed between UT (−90.1 ± 24.000) and WT horses (27.2 ± 8.130) (Figure 6C).

An opposite impact in the concentration of anti-inflammatory cytokines was detected. A significant increase in IL-4 concentration after exercise was observed only in WT horses (*p* = 0.0078; 19.9 ± 4.370 vs. 33.5 ± 2.090) but not in UT horses (*p* = 0.0977; 45.4 ± 7.280 vs. 34.4 ± 2.880) (Supplementary Figure S5B). A decrease of IL-10 concentration after exercise in UT (*p* = 0.0039; 574.0 ± 74.500 vs. 187.0 ± 21.400) and an increase in WT (*p* = 0.0039; 139.0 ± 48.000 vs. 263 ± 51.400) horses were observed (Supplementary Figure S5D). Significant differences in IL-4 (*p* = 0.0007; −11.0 ± 5.120 vs. 13.6 ± 2.790) and IL-10 (*p* < 0.0001; −388.0 ± 73.200 vs. 124.0 ± 35.800) concentrations were noted between UT vs. WT horses (Figure 6B,D).

## 4. Discussion

### 4.1. Lymphocytes Proliferation

In human studies the influence of exercise on the immune system response in the form of lymphocyte proliferative activity is often measured [19]. However, it is debatable whether lymphocyte proliferation is impaired or not during exercise. It was postulated that the influence of physical activity is duration- and intensity-dependent. In one study, 50% and 25% reductions in lymphocyte proliferation after 45 min of treadmill running at 80% and 50% VO_2_max, respectively, were observed in humans [20]. However, in a more recent study after 30 min of running at 80% or 60% VO_2_peak, the proliferation was not affected in sportsmen [21]. In humans, a recent meta-analysis of 24 studies showed that lymphocyte proliferation is inhibited following acute physical training lasting longer than 1 h, regardless of exercise intensity [22]. In this study, an increased lymphocyte proliferation in WT horses was noted. Nevertheless, decreased or unchanged lymphoproliferative responses following treadmill exercise and racing in horses have been found [23,24]. However, in both of these studies only concanavalin A (Con A), phytohemagglutinin (PHA), or pokeweed mitogen (PWM) were used. Inhibited proliferation upon mitogen stimulation indicates that the NK cell numbers increase more than the T cell counts during exercise [19]. Thus, decreased response to PHA and Con-A reflects only a reduced fraction of CD4+ or CD8+ cells which was not observed in this study. In addition, the stimulation was supplemented with IL-2, and the cell culturing lasted 4 days. Whereas, in studies by Nesse et al., 2002 and Kurcz et al., 1988 [23,24], the stimulation took only 2 h and 48 h, respectively. This may be too short for proper cell division, because in humans, peak mitogenic responses are shown after at least 72 h or even 4–5 days culturing [25]. Furthermore, as was mentioned earlier in our study the exercise did not last longer than 1 h.

In UT horses the proliferative response decreased after acute exercise. This is in line with other studies that have shown that unconditioned horses exhibit decreased lymphoproliferative responses to mitogens and antigen-specific stimulation during intensive exercise [26,27]. However, following conditioning, the proliferative activity increases [19,28,29], which is consistent with the present study’s findings in WT horses.

### 4.2. T Lymphocytes

Mature T regulatory lymphocytes express CD4+CD25+ in humans. However, in horses, due to lack of the equine specific CD25 antibodies and relatively low homology of the equine CD25 gene, CD4+FoxP_3_+ cells are also considered as Tregs [30]. In addition, in horses as in humans, only CD4+ CD25high cells are FoxP_3_+, making equids a good model for the examination of those cells in various diseases [31].

In WT horses the number of CD4+ and CD4+FoxP_3_+ cells increased after race training in contrast to inexperienced animals. This is in line with human studies performed on sprint-trained, elite swimmers and endurance-trained athletes [32,33,34]. In equids, the decreased number of CD4+ cells after long-distance low-intensity training was documented but only 3 horses participated in that endurance ride [35]. High-intensity exercise may decrease the number of CD4+ cells [13,36]. However, it is speculated that it may depend not only on the type of exercise but also on the fitness level of the athlete. In this study, in UT horses there was a decrease in CD4+ cells. In addition, there was an increase in CD8+ cytotoxic T cells. In a related study using untrained equids subjects, an acute bout of exercise resulted in a decrease in the percentages of CD4+ and an increase in the percentages of CD8+ cells as well [37]. However, after 12-weeks of conditioning these horses had lower percentages of CD8+ cells in a post-simulated race test.

Despite the lack of other studies in horses, it was documented that both regular race training and endurance training lead to an anti-inflammatory state [16,38]. However, in these studies, only the cytokines’ response was measured. The expression of FoxP_3_ on lymphocytes CD4+ correlates with their controlling and suppressor function both in humans and equids [31,39]. This effect in humans is mostly connected with higher IL-10 secretion associated with increases in the numbers of circulating Treg cells [39]. An increased level of this cytokine, of strong anti-inflammatory action, was reported after exercise in elite endurance horses [40]. In the present study, an increase in CD4+ FoxP_3_, and CD8+ FoxP_3_ was observed only in WT horses, which may be connected with better conditioning and the creation of an anti-inflammatory state in those animals. In addition, it can be a time-dependent change related with the lymphocyte redistribution to peripheral tissues. In humans, it was documented that T-regulatory cells exhibit a biphasic response to prolonged, highly strenuous exercise [41]. In the early hours of recovery from a marathon, a decrease in the absolute number of CD3+CD4+ lymphocytes occurred, whereas after one day their number increased. Unfortunately, we were not able to measure the cell response at later time points because the samples were collected during the standard periodic veterinary procedure.

Another possible reason for the differences between the percentages of CD4+FoxP_3_+ and CD8+ FoxP_3_ cells in trained and untrained horses may be age related. In foals, the significantly enhanced suppressive capability of FoxP_3_+ cells was noted compared to their mothers and yearlings [42]. Thus, in untrained 2–3 year-old thoroughbreds the lower proportion of FoxP_3_+ cells after exercise may be to balance higher immunosuppressive action than in 3–4 year-old trained horses.

Thus, the present study confirmed that physical exercise initiates the change in the immune system functionality. In UT horses the high increase of T cytotoxic lymphocyte numbers (CD8+) was probably a consequence of tendon and muscle microinjury. However, the inflammatory process may lead to musculoskeletal system remodeling following adaptation to increasing workload during the training process. The results obtained in WT horses with the increased percentages of T regulatory cells (CD4+FoxP3+ and CD8+FoxP3+) confirmed that creation of immunosuppressive conditions is a mode of adaptation of horses to training. It could protect these animals from musculoskeletal system injury-associated inflammation.

### 4.3. Monocytes

Monocytes exhibit highly pleiotropic functions. They are responsible for the presentation of foreign antigens to T cells, phagocytosis, and secreting cytokines and chemokines of various properties [43]. In humans, blood monocytes are classified as classical (CD14++CD16−), non-classical (CD14+CD16++), and intermediate cells (CD14+CD16+) [44]. The classical and intermediate monocytes are considered pro-inflammatory cells, whereas the non-classical ones have an anti-inflammatory action [45]. An increased number of monocytes with inflammatory properties was observed after a single bout of strenuous exercise in humans [46]. However, there is inconsistency in results because in other studies it was shown that acute exercise did not modify the innate immune responses [47]. In addition, regular physical activity lowers pro-inflammatory monocyte properties [47,48].

There are only a few studies concerning equine monocytes because of the low availability of species-specific monoclonal antibodies [49,50,51]. Furthermore, there is a lack of studies connected with the influence of exercise on monocyte function in equids. Thus, it was hypothesized that CD14–MHCII+ monocytes are equivalent to human non-classical monocytes, while CD14+MHCII+ represent intermediate and CD14+MHCII− are classical monocytes, the same as in dogs [52,53]. In horses, an increase in the percentage of CD14+ cells in LPS (Lipopolisacharide)-stimulated individuals occurred [51]. Furthermore, the downregulation of MHCII expression on immune cells was still present 4 h after endotoxin infusion [51]. Thus, CD14+MHCII− monocytes may also have a pro-inflammatory action in equids. 

In this study, there was was a post-exercise increase of CD14–MHCII+ and no change in CD14+MHCII− in WT horses, in contrast to UT individuals, in which an increase of CD14+MHCII− and a decrease of CD14–MHCII+ monocytes occurred. Thus, the percentage of cells with an anti-inflammatory action was increasing during the conditioning. However, a lower number of anti-inflammatory monocytes in the bloodstream could be also caused by increased migration of these cells to the peripheral tissues. Skeletal muscle regeneration and differentiation are modulated by the local inflammatory process [54,55]. Thus, these cells may regulate the inflammatory response in the muscles, and their remodeling. Unfortunately, no muscle biopsy was performed due to invasiveness. However, age-related differences exist. In foals, the activation of peripheral blood monocytes results in more robust production of IL-10 compared to adult horses [56]. Thus, reduction in CD14–MHCII+ in UT horses may be a compensative reaction preventing immunosuppression. However, in the study, such differences were relatively marginal (2–3 vs. 3–4 years old).

The exact role of CD14+MHCII− and CD14–MHCII+ equine monocytes is unclear. Due to differences between species and the inability to identify the same markers as in humans, direct comparisons still leave a lot of uncertainty. Further analysis and functional studies are needed to confirm that regular exercise reduces the numbers of pro-inflammatory monocytes in horses.

### 4.4. Reactive Oxygen Species Production

Exercise-induced increase in the generation of reactive oxygen species (ROS), including free radicals, has been demonstrated, especially after exhaustive physical activity both in humans [57,58,59] and horses [60]. Although oxidants can be produced in a variety of tissues, most of them are derived from skeletal muscle contraction [61]. However, non-muscular cells also respond to exercise-associated redox-sensitive signaling effects. ROS production is one of the monocytes’ prominent functions, which allows them to modulate the function of other immune cells [62]. In the present study, the increased ROS production by CD14+ cells after exercise was documented in both WT and UT horses. However, the elevation in the ROS production was higher in the UT thoroughbreds. In addition, the ROS level in CD5+ cells was only increased in UT horses. The differences may be caused by adaptational processes which lead to a decrease in ROS production during training [61]. This prevents extensive tissue damage connected with oxidative stress. However, the transient oxidative stress can generate ligands that activate anti-inflammatory signaling systems such as Peroxisome Proliferator-Activated Receptor-gamma (PPARγ) and Liver X-Receptor-alpha (LXRα) in monocyte-macrophages during training [63]. In addition, ROS modulate macrophages’ immunosuppressive phenotype through the up-regulation of programmed death-ligand 1 (PD-L1) [64]. Thus, more ROS production at the beginning of the race training may enhance the creation of the anti-inflammatory state.

### 4.5. Cytokine Production

In this study, the proinflammatory cytokine production by PBMCs such as IL-1β, IL-17, and TNF-α was only increased in UT horses after physical activity. It was documented that high-intensity exercise results in a transient increase in the expression of cytokines such as IFN-γ, IL-1, and TNF- α in the horses’ muscles and blood [16,65]. Furthermore, in endurance horses cytokines type 1 (IL-1β and TNF-α) were elevated in the bloodstream at the beginning of the training process [38]. In addition, the production of cytokines (IL-6 and IL-10) with anti-inflammatory properties was reduced in UT horses after exercise. The decreased production of IL-10 may be explicable by the decreased percentage of Tregs [39]. IL-6 is a cytokine of both pro- and anti-inflammatory action, depending on the signaling type (classical vs. trans-signalization) [66]. The plasma concentration of IL-6 in response to acute exercise increases more than any other cytokines [16,38,65]. However, the main source of this protein is skeletal muscle [67]. In this study, only cytokines’ production by PBMCs was measured. Thus, it cannot be compared directly with results obtained from blood collected after exercise. However, there is a lack of studies that measure the ability of the cells to produce cytokines after exercise in horses [65].

In contrast, in WT racehorses, there was an increase in anti-inflammatory cytokines IL-4, IL-6, and IL-10, and a decrease of those with pro-inflammatory properties (IL-17, INF-γ). It was documented that IL-4, IL-6, and IL-10 are involved in the suppression of LPS-induced TNF-α and IL-1 production [68,69]. This is in line with other studies in which regular physical activity results in the decrease of pro-inflammatory states [70].

In addition, this is the first study measuring changes in IL-17 production during race training in horses. Surprisingly, the production of this cytokine after exercise was lower in WT horses, whereas in UT animals its level was up-regulated. In humans, it is well known that IL-17 mediates inflammatory and tissue remodeling events in early tendinopathy [71]. Thus, in UT horses this cytokine may play a crucial role during tendon adaptation to physical activity. In WT horses the process of tendon adaptation may be completed. In contrast, to prevent tendinopathy the high production of IL-17 by PBMCs is limited, especially when IL-6 and IL-10 production is up-regulated. Early human tendon healing is characterized by higher production of IL-6, IL-8, and IL-10 [72]. In addition, the results suggest that the PBMCs are a very important source of IL-17 in racehorses. However, more research is needed to support this thesis and to confirm if there are other sources of this peptide.

### 4.6. Main Limitations

The main limitation of the study is the relatively low number of horses. In addition, the blood samples were collected exclusively during a single standard veterinary procedure. This prevented checking the time-dependent cell reaction to exercise especially during the recovery period. In addition, a muscle biopsy would have provided additional information, but this was not performed. Moreover, because of the low availability of equine specific antibodies, more CD markers to confirm a more precise role of the individual cell population are needed. Moreover, another limitation may be the influence of the age of the horses because it might have been somewhat confounded with the training level. UT horses were a slightly younger (2–3 years old) whereas the WT horses were slightly older (3–4 years old). However, the difference is still quite marginal. The main decrease in immunological system activity was noted in foals (median age 3.5 months), whereas only a slight difference existed between yearlings and adult horses (12–26 years old) [42].

## 5. Conclusions

In general, the immune response to acute exercise is transitory and variable. The study confirmed that during conditioning the immune system also adapts. The cells from highly trained racehorses polarize to an anti-inflammatory phenotype, whereas in young horses more pro-inflammatory reactions occurred. Thus, more attention should be paid to exercise testing especially at the beginning of race training. Finally, it should be noted that cell response in the future may be used as a novel diagnostic “biomarker” for exercise-induced adaptational change as circulating cells are relatively easy to collect with low invasiveness. However, more studies are required to establish the biological significance of immunological system reactions due to physical activity.

## Figures and Tables

**Table 1:** List of monoclonal antibodies used for labeling peripheral blood mononuclear cells (PBMCs) for flow cytometry.

Antibody	Clone; Dilution	Source	Target Cell
CD4:PE	CVS4; 1:10	BioRad, Berkeley, CA, USA	lymphocytes
CD8:FITC	CVS21; 1:10	BioRad, Berkeley, CA, USA	lymphocytes
CD5:PE	CVS5; 1:10	BioRad, Berkeley, CA, USA	lymphocytes
CD14:AF405	433423; 1:10	R&D Systems, Minneapolis, MI, USA	monocytes
MHCII:FITC	CVS20; 1:20	BioRad, Berkeley, CA, USA	monocytes
FoxP3:APC	FJK-16s; 1:10	Life Technologies, Bleiswijk, The Netherland;	lymphocytes

## Supplementary Materials

The following are available online at https://www.mdpi.com/2076-3921/9/11/1155/s1, Figure S1: Graphs showing the change of ex vivo proliferation rate of (A) total, (B) CD4+, and (C) CD8+ lymphocytes under exercise obtained from both untrained and well-trained horses. Figure S2: Graphs showing the change in the percentage of T lymphocytes such as: (A) CD4+, (B) CD8+, (C) CD4+FoxP3+, and (D) CD8+FoxP3+ under exercise obtained from both untrained and well-trained horses. Figure S3: Graphs showing the change in the percentage of monocytes such as: (A) CD14-MHCII+, (B) CD14+MHCII-, and (C) CD14+MHII+ under exercise obtained from both untrained and well-trained horses. Figure S4: Graphs showing the change in percentage of positive cells: CD14+CR+ (A), CD5+CR+ (B), and median fluorescent intensity of CR in CD14+ (C) and CD5+ (D) cells under exercise obtained from both untrained and well-trained horses. Figure S5: Graphs showing the change of cytokine concentrations: IL-1β (A), IL-4 (B), IL-6 (C), IL-10 (D), IL-17 (E), TNF-α (F), and IFN-γ (G) under exercise, presented in a culture medium of PBMCs obtained from untrained and well-trained horses. All data are presented as the mean ± SEM. Significance levels: * *p* < 0.05; ** *p* < 0.01; *** *p* < 0.001.
