# Supplementary material for: The Effect of Physical Training on Peripheral Blood Mononuclear Cell Ex Vivo Proliferation, Differentiation, Activity, and Reactive Oxygen Species Production in Racehorses
Source: Antioxidants (Basel). 2020 Nov 20;9(11):1155. doi: 10.3390/antiox9111155 (PMC7699811; doi:10.3390/antiox9111155)
Supplement: Supplementary file 1 [file antioxidants-09-01155-s001.pdf]

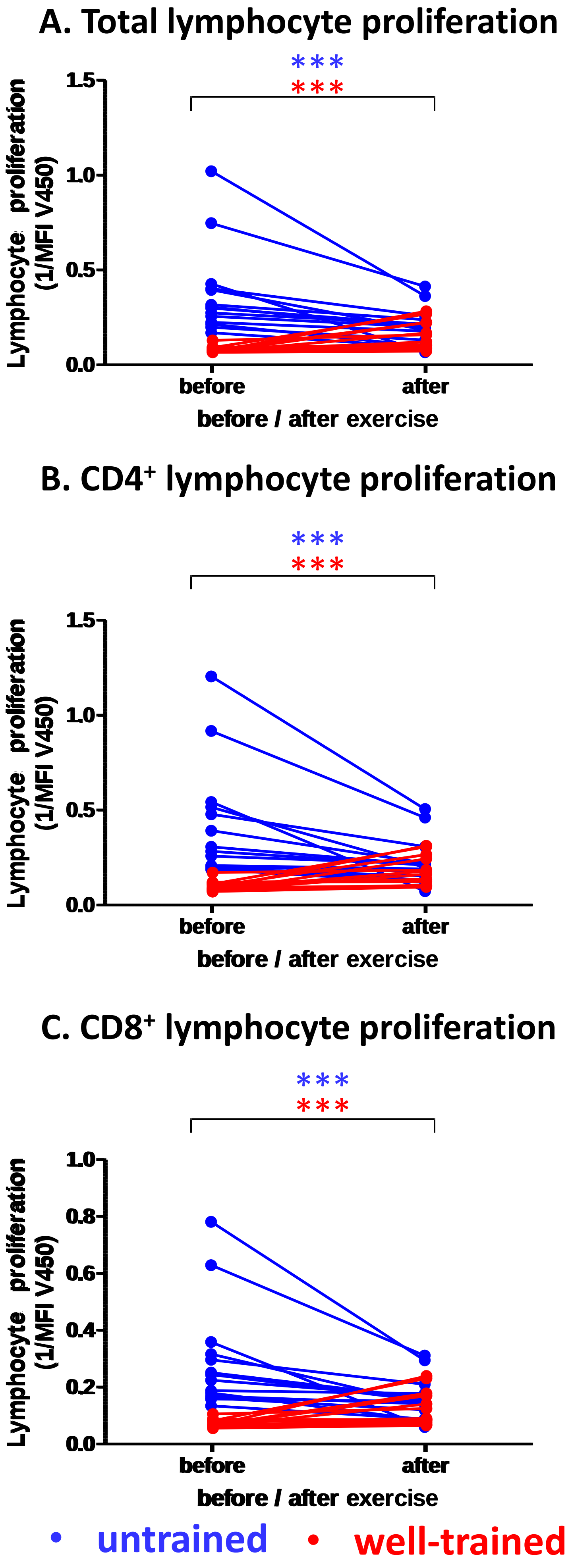

Supplementary Fig. 1.

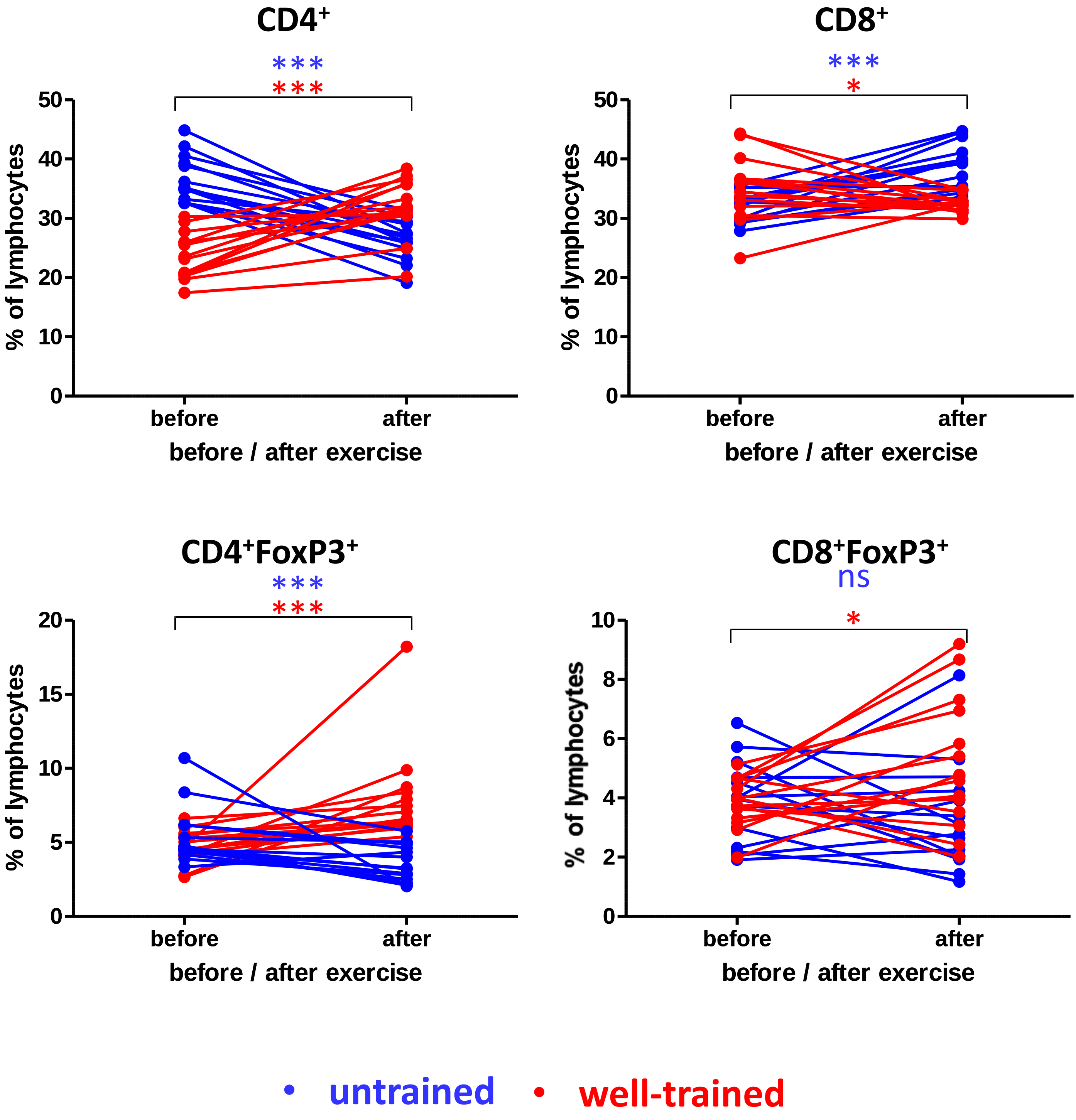

Supplementary Fig. 2.

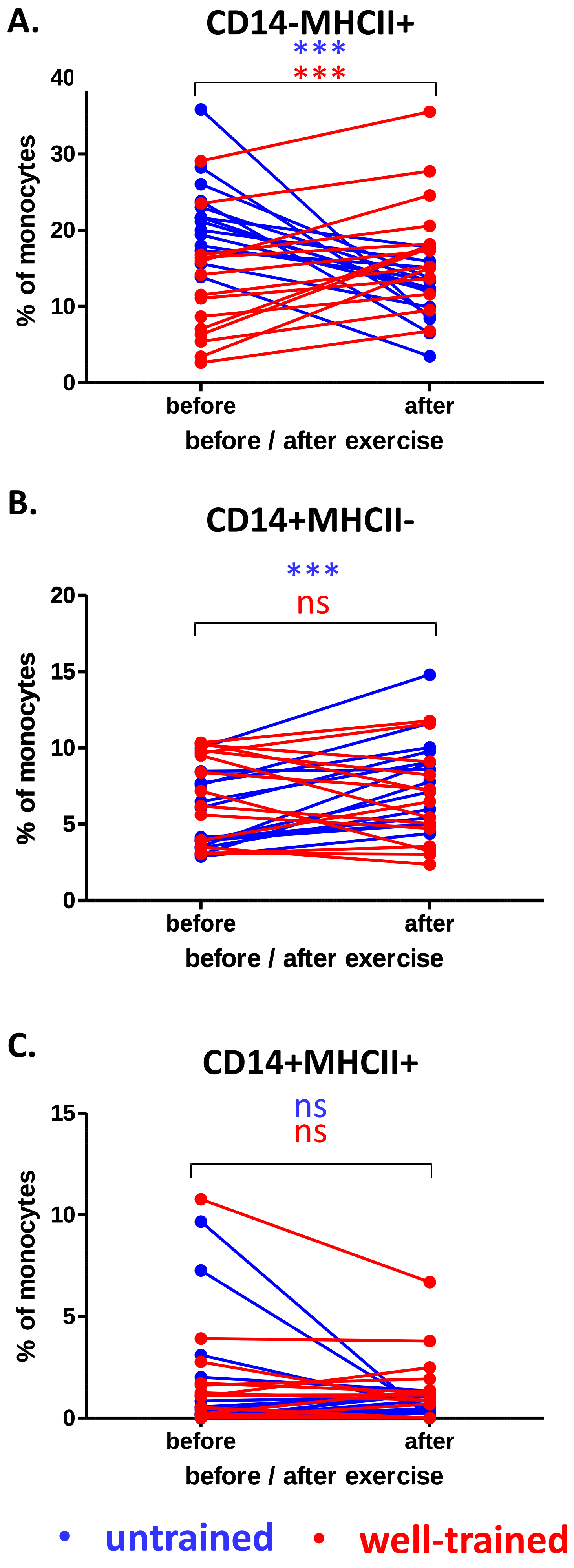

Supplementary Fig. 3.

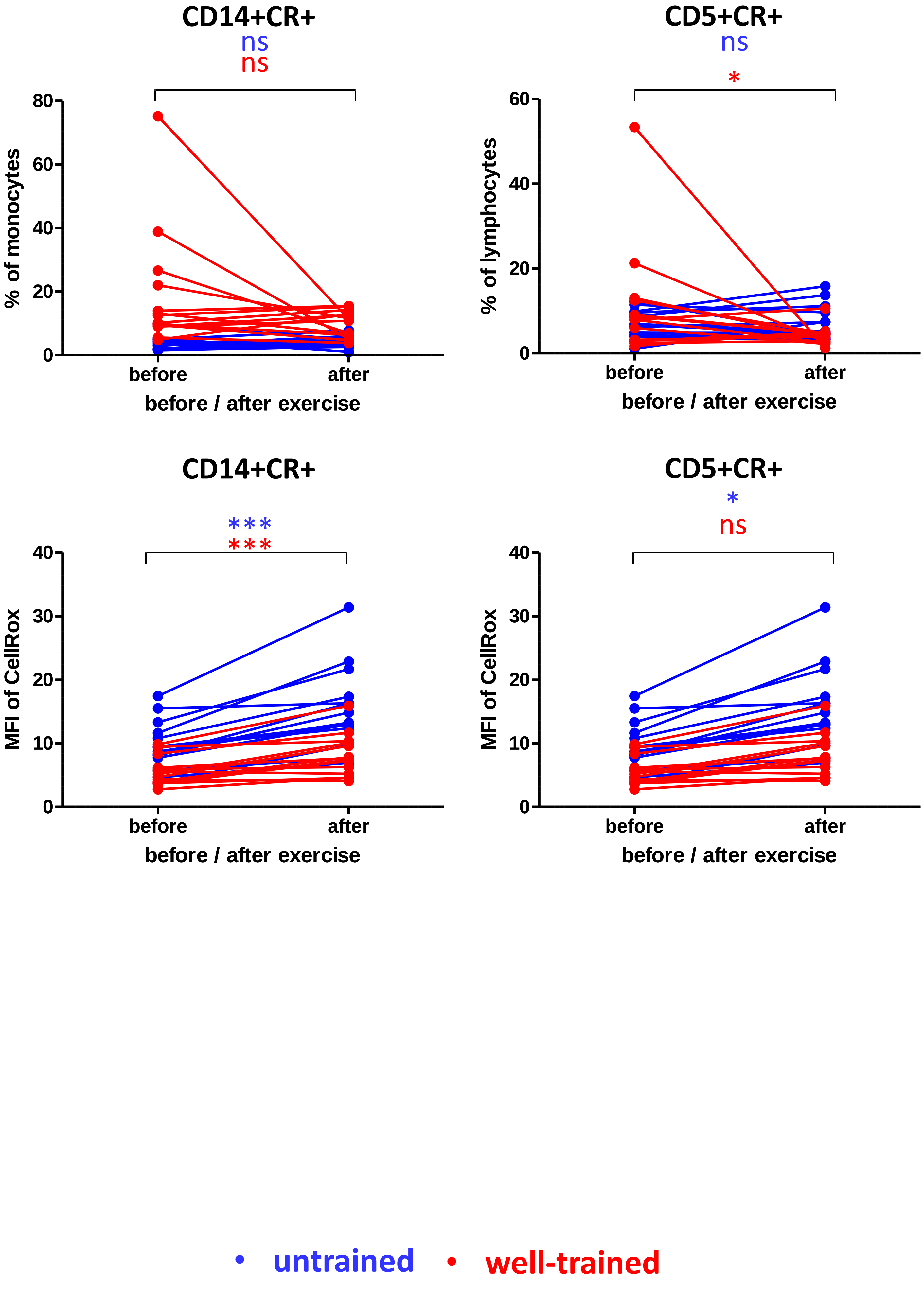

Supplementary Fig. 4.

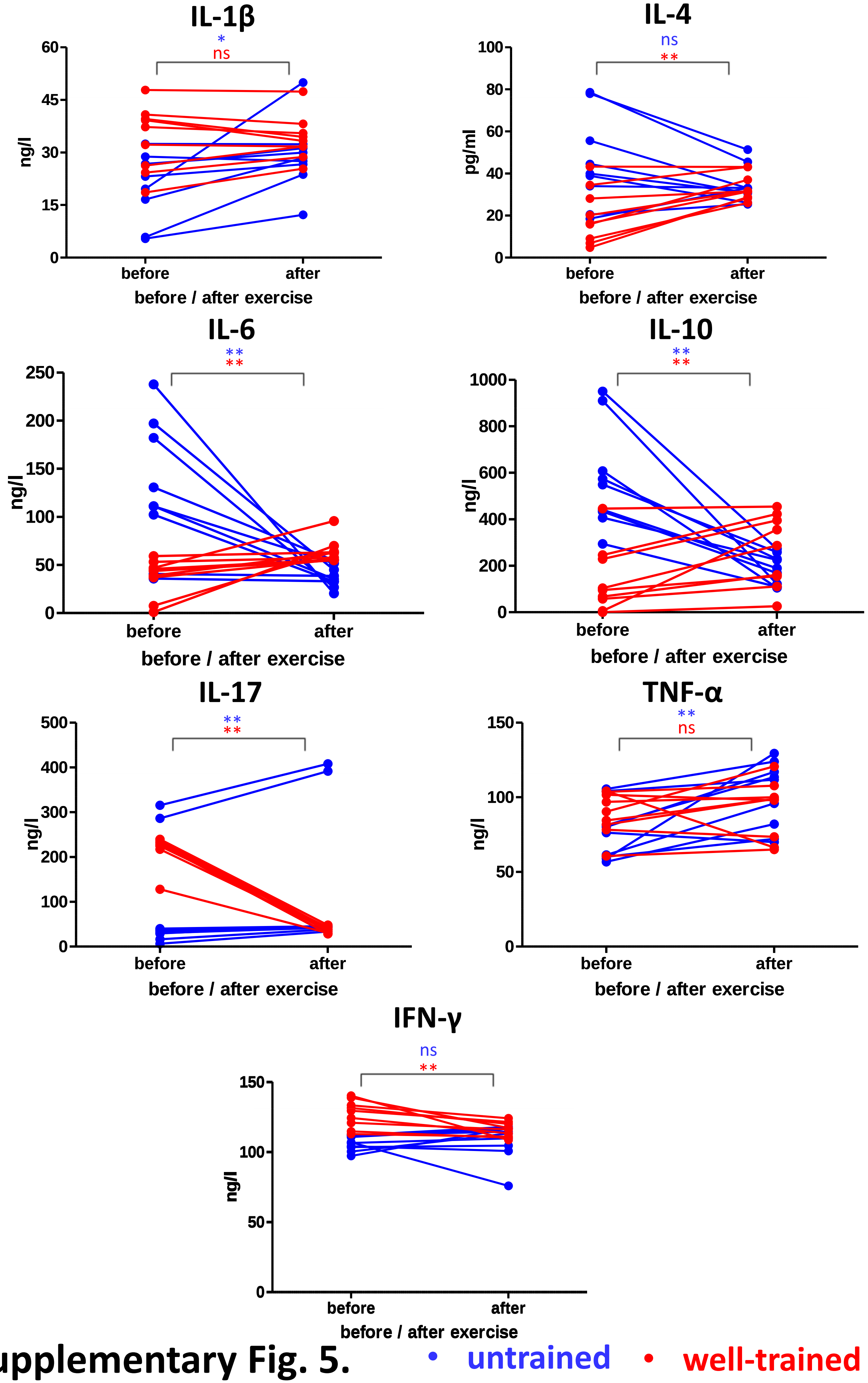

Supplementary Fig. 5.
